# Molecular Identification and Susceptibility of Clinically Relevant *Scedosporium* spp. in China

**DOI:** 10.1155/2015/109656

**Published:** 2015-10-07

**Authors:** Hong Wang, Zhe Wan, Ruoyu Li, Qiaoyun Lu, Jin Yu

**Affiliations:** ^1^Department of Dermatology and Venereology, Peking University First Hospital, Research Center for Medical Mycology, Peking University, Beijing 100034, China; ^2^Beijing Key Laboratory of Molecular Diagnosis of Dermatoses, Beijing 100034, China; ^3^Department of Dermatology, Central Hospital of Xiangyang, Hubei College of Arts and Science, Hubei 441021, China

## Abstract

As various new sibling species within the *Scedosporium* spp. have been described recently, this study was conducted to investigate distribution and antifungal susceptibility profiles of the different species of *Scedosporium* spp. in China. Twenty-one clinical strains of *Scedosporium* from China and two strains from Japan were reidentified by MLSA. The analysis included BT2, CAL, RPB, SOD, and ACT and the combination of the five loci. *Pseudallescheria boydii* complex (17 strains) and *S. apiospermum* (6 strains) were identified. *P. boydii* complex included four closely related subgroups: *P. boydii* (9 strains), *P. ellipsoidea* (6 strains), *P. fusoidea* (1 strain), and *P. angusta* (1 strain). There were no significant differences in MICs for neither VOR, POS, nor AMB over all the five species in study. For itraconazole, intraspecific diversity was evident.

## 1. Introduction

*Scedosporium* spp. is one of the species of the opportunistic pathogenic fungi that can always be found in environment, especially in sewerage. It infects immunocompromised patients and drowning men, involving lungs, sinuses, bones, joints, eyes, and brain [1].

In recent years, members of the genus* Scedosporium* are increasingly recognized as opportunistic agents of disease, for example, in transplant recipients.* Scedosporium* species have been identified as the second most prevalent mold after* Aspergillus* colonizing the lungs of patients with cystic fibrosis [2].* Scedosporium* infections occur worldwide. In European countries, USA and Australia,* Scedosporium* species were always found in patients with chronic lung diseases, cystic fibrosis (CF), lung or allogenic bone-marrow transplantation, and hematologic malignancies [1, 3, 4]. Accordingly, the common types were pulmonary, nasal sinuses, skin/soft tissues, CNS, and disseminated infection [1, 3, 4].

It is traditionally recognized that* Pseudallescheria boydii* is the sexual stage of the* S. apiospermum*. However, recent molecular studies [5–7] showed that* Scedosporium* is a species complex comprising at least five distinct groups:* S. aurantiacum*,* P. minutispora*,* S. dehoogii*,* S. apiospermum*, and* P. boydii*, the last group consisting of four closely related subgroups called* P. boydii*,* P. angusta*,* P. ellipsoidea*, and* P. fusoidea.* Furthermore,* S. prolificans* is now renamed as* Lomentospora prolificans* [8]. It is important to identify* Scedosporium* spp. to species level because their virulence, metabolic trait, and* in vitro* susceptibility may be various based on their different species [3–9].

## 2. Materials and Methods

### 2.1. Strains

From 1990 to 2014, twenty-one* Scedosporium* strains isolated from patients were reserved in Research Center for Medical Mycology at Peking University. The clinical samples were collected from 14 Chinese hospitals which located mainly in central and south of China. All the isolates were identified as* S. apiospermum* or* P. boydii* by morphology. A total of 23 isolates (including two strains from Japan) as shown in Table 1 were investigated in this study. Furthermore,* in vitro* susceptibility was performed on the same set strains.

### 2.2. Molecular Studies

The isolates were cultured on PDA at 28°C for 7 days. For fungal DNA extraction, glass beads method previously described by van Burik et al. was followed [10]. Adapted from earlier genotyping studies [11–14], PCR amplification with different primer pairs was attempted for* Scedosporium* species for the following genes: *β*-tubulin (BT2, exons 2–4) [11], calmodulin (CAL, exons 3–4) [12], the second largest subunit of RNA polymerase II (RPB) [13], superoxide dismutase (SOD), and actin (ACT) [14].

The PCR assay (25 *μ*L) included 2 *μ*L of fungal DNA extract, 1 *μ*M of each gene-specific primer, 2.5 mmol dNTP Mix 1 *μ*L, 10x PCR buffer 2.5 *μ*L, and LA Taq polymerase 0.25 *μ*L (Fermentas, St. Leon-Rot, Germany). The amplification for all targeted genes was performed in a Eppendorf PCR machine (AG22331) as follows: 5 min of initial denaturation at 95°C, followed by 35 cycles at 95°C for 30 s, gene-specific annealing temperature for 30 s, and 72°C for 1 min (for RPB2 the annealing time was 2.5 min). The PCR products were visualized by electrophoresis on a 1% (w/v) agarose gel. Both strands of the PCR fragments were sequenced using the above-mentioned primers. The consensus sequences were obtained using SeqMan (DNAStar-Lasergene, Madison, WI, USA) software. Newly obtained sequences were deposited in GenBank under accession numbers KP 981107 to KP 981221 (Table 1). They were used to conduct alignment analysis for preliminary species identification in the NCBI genomic database (http://blast.ncbi.nlm.nih.gov/) and CBS database (http://www.cbs.knaw.nl/). The sequences were aligned using MUSCLE. For the maximum likelihood analysis, the distances between sequences were calculated using the best parameter model found by MEGA 6.0 6 (http://www.megasoftware.net/). A bootstrap analysis was conducted with 1000 replications.

### 2.3. Susceptibility Test

The* in vitro* susceptibility of the 23* Scedosporium* isolates against four antifungal agents was evaluated by using the Clinical and Laboratory Standards Institute (CLSI) M38-A2 broth microdilution method [15]. The inocula suspensions were prepared in new sterile tubes and adjusted to 0.4−5 × 10^6^ colony-forming units per milliliter (CFU/mL) by counting spores in a hemocytometer and subsequently verifying them through quantitative colony counts on PDA plates. The nongerminated spore suspensions were diluted 1 : 100 in an RPMI 1640 to achieve a final inoculum concentration of 0.4–5 × 10^4^ CFU/mL. The following antifungal agents were used: voriconazole (VOR; Shouguang Fukang Pharmaceutical Co., Ltd., China), posaconazole (POS; Merck, Rahway, NJ, USA), itraconazole (ITR; Shouguang Pharm), and amphotericin B (AMB; Sigma-Aldrich Co., St. Louis, USA). They were all diluted in 100% dimethyl sulphoxide as a stock solution with a concentration of 1.600 mg/L. Final drug concentrations ranged from 16 to 0.03 mg/L for all the four drugs. The minimal inhibitory concentrations (MIC) endpoints were defined as the lowest concentration at which there was a complete inhibition of growth.* Aspergillus flavus* ATCC 204304 served as a quality control strain. The microtiter panels were incubated at 35°C and the results were read after 72 h. All tests were performed in triplicate on three different days.

## 3. Results

### 3.1. Molecular Phylogeny

**Figure 1:**
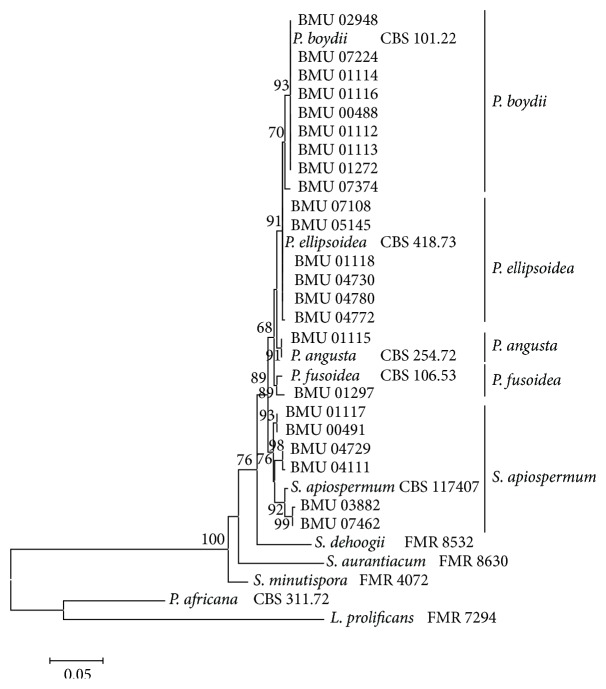
Maximum likelihood tree based on BT2 sequences. Bootstrap values of >50% are indicated ion branches. The bar indicates the number of substitutions per site.

**Figure 2 fig2:**
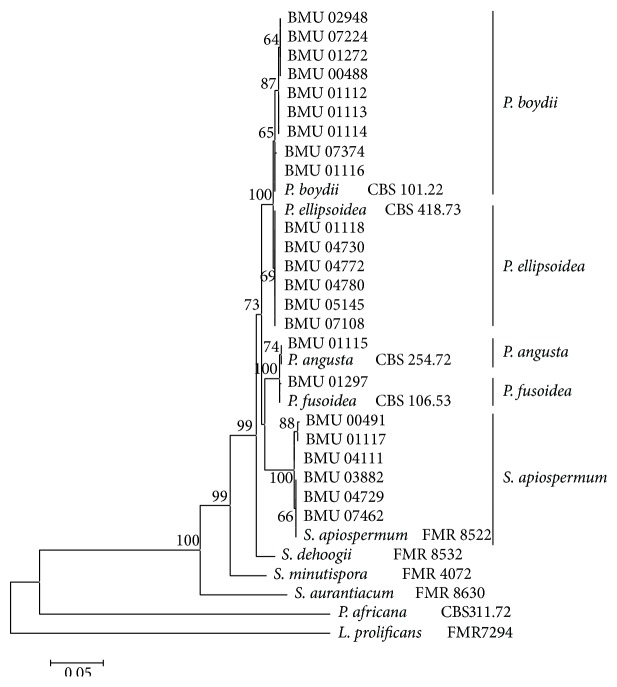
Maximum likelihood tree based on CAL sequences. Bootstrap values of >50% are indicated ion branches. The bar indicates the number of substitutions per site.

**Figure 3 fig3:**
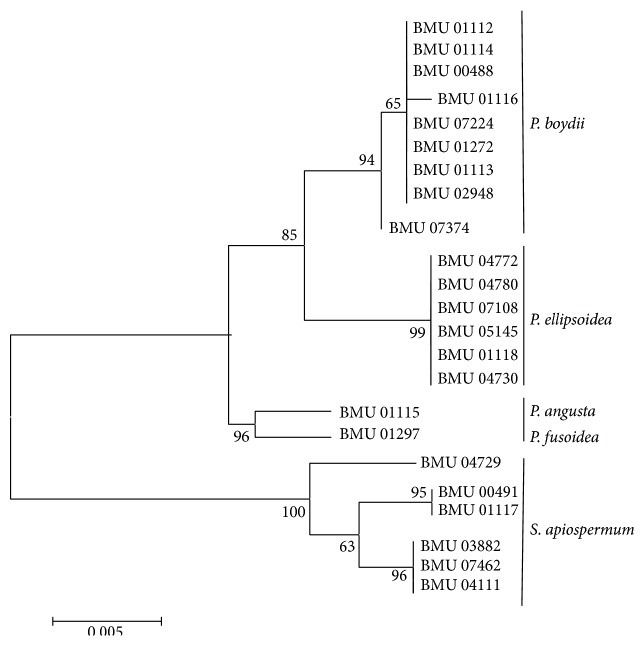
Maximum likelihood tree based on RPB. Bootstrap values of >50% are indicated ion branches. The bar indicates the number of substitutions per site.

**Figure 4 fig4:**
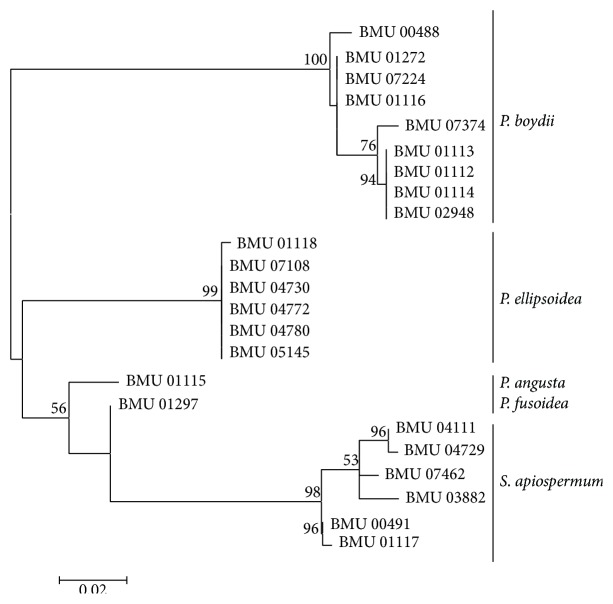
Maximum likelihood tree based on SOD sequences. Bootstrap values of >50% are indicated ion branches. The bar indicates the number of substitutions per site.

**Figure 5:**
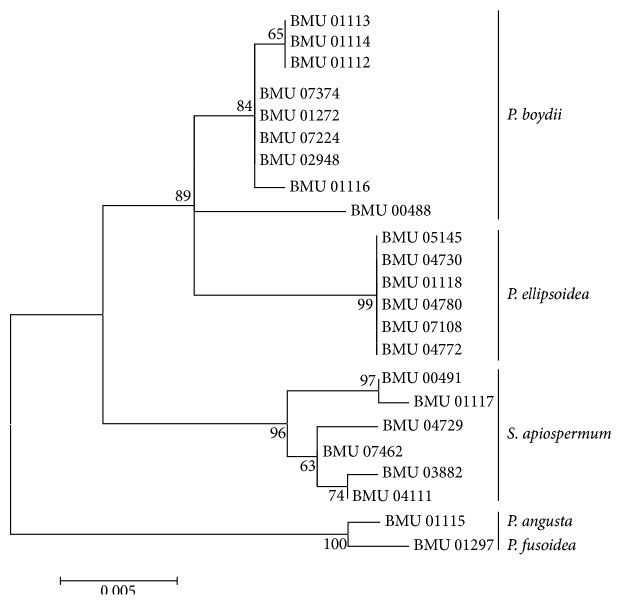
Maximum likelihood tree based on ACT sequences. Bootstrap values of >50% are indicated ion branches. The bar indicates the number of substitutions per site.

**Figure 6:**
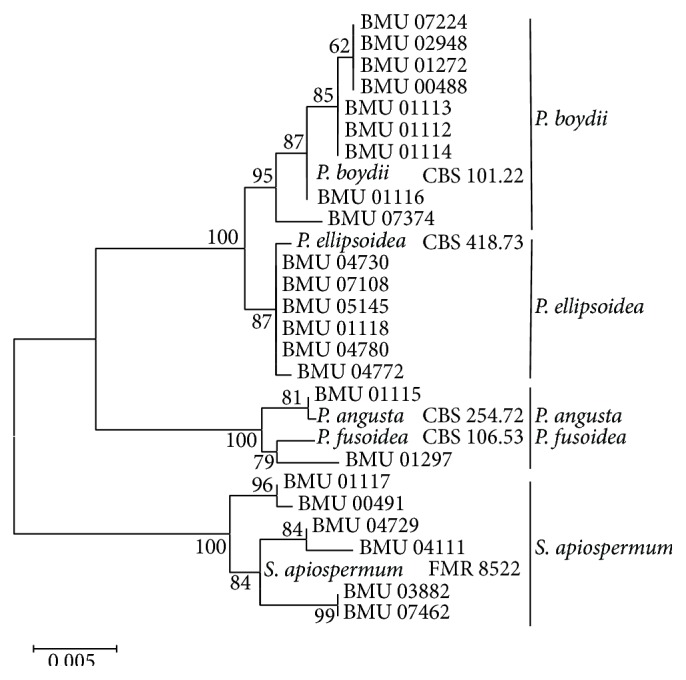
Maximum likelihood tree based on the analysis produced from the combined BT2 and CAL data. Bootstrap values of >50% are indicated ion branches. The bar indicates the number of substitutions per site.

We were able to amplify and sequence 470 bp, 645 bp, 935 bp, 775 bp, and 396 bp of the BT2, CAL, RPB, ACT, and SOD loci, respectively. Of the 3221 nucleotides sequenced, 209 (6.5%) were informative for parsimony in the different* Scedosporium* isolates. For identification, reference sequence of* Scedosporium* species available in public database were used including BT2 and CAL, but no sequences for RPB, SOD, and ACT were available. The sequences for BT2, CAL, RPB, SOD, and ACT yielded phylogenetic trees with the same topology (Figures 1 [Fig fig2] [Fig fig3] [Fig fig4]–5). In the combination phylogenetic trees based on BT2 and CAL (Figure 6), 23 strains in our study were reidentified to the species level according to the reference strains, which were analyzed in the Gilgado literature.* P. boydii* (9/23) and its closely related subtypes* P. ellipsoidea* (6/23),* P. fusoidea* (1/23), and* P. angusta* (1/23) were the most common, and the other 6 of 23 strains were identified as* S. apiospermum*.

**Figure 7:**
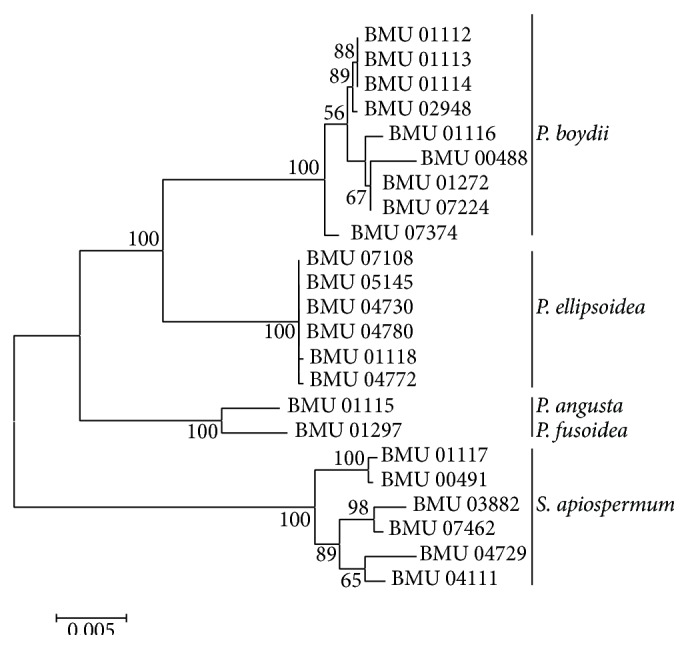
Maximum likelihood tree based on the analysis produced from the combined all five markers data. Bootstrap values of >50% are indicated ion branches. The bar indicates the number of substitutions per site.

The topology of the combined tree (Figure 7) of all five loci was similar to those observed in the trees of individual locus and the combined tree of BT2 and CAL. Four principal clades were obtained. The four clades were the* P. boydii* clade,* P. ellipsoidea* clade,* P. fusoidea*/*P. angusta* clade, and* S. apiospermum* clade.* P. fusoidea* and* P. angusta* always assemble together, and* P. boydii* and* P. ellipsoidea* were very closely related.* S. apiospermum* has the highest intraspecies variability (genetic distance = 0.008), which is comparable to the interspecies variability between* P. fusoidea* and* P. angusta* (genetic distance = 0.008).

### 3.2. *In Vitro* Susceptibility Test

The MIC values for the four antifungal agents examined by the CLSI M-38A2 microdilution method against the 23 strains are presented in Table 2. VOR was the most active agent against all 23 strains with a MIC range from 0.25 to 1 *μ*g/mL and a 0.46 *μ*g/mL GM. POS was the second most active agent with MIC values of 2 or 4 *μ*g/mL. For ITR, the intraspecific diversity was obvious as most strains had a MIC between 2 and 4 *μ*g/mL, but five strains were higher at a MIC of 32 *μ*g/mL. AMB is the least effective with high MIC values ranging from 4 to 32 *μ*g/mL* in vitro*.

The MIC_50_ and MIC_90_ for the four antifungal agents against* P. boydii*,* P. ellipsoidea*, and* S. apiospermum* are presented in Table 3. There were similar MIC_50_ and MIC_90_ for VOR, POS, or AMB of the three species above. For ITR, the MIC_90_ of* P. boydii* (4 *μ*g/mL) was lower than that of* P. ellipsoidea* (32 *μ*g/mL) and* S. apiospermum* (32 *μ*g/mL).

## 4. Discussions and Literature Review

Scedosporiosis is a rare infection in China. Sporadic cases of* Scedosporium* infection have been reported since 1990, but most cases were identified in recent years. We reviewed 39 cases reported in China from 1990 to 2013 [16–31]. We found that infection in the nasal sinuses (16 cases) was the most frequently involved location, followed by eye (13), CNS (5), lung (4), skin/soft tissues (2), arthritis or osteomyelitis (1), and disseminated infection (1). The risk factors included trauma, drowning, and a compromised immune system (bone marrow transplant or stem cell transplantation). Trauma is the most common risk factor for scedosporiosis in Chinese patients, as nearly half of these patients have trauma history. Because cystic fibrosis is extremely rare in the Chinese population, no scedosporiosis in CF was reported in China.

Based on molecular identification, we found that* P. boydii* complex and* S. apiospermum* were the only two species in our study. Actually,* P. boydii* and* S. apiospermum* demonstrated a closely phylogenetic relationship. This is also reflected by their undifferentiated morphological characteristics, assimilation of different sugars, and growth temperature (data not shown). In* P. boydii* complex, four closely related subgroups,* P. boydii* (9 strains),* P. ellipsoidea* (6 strains),* P. fusoidea* (1 strain), and* P. angusta* (1 strain), were present in the study.

In a set of clinical and environmental strains from Austria, Germany, and Netherlands,* S. apiospermum* was the most prevalent, followed by* P. boydii* [32, 33]. In Australia,* S. apiospermum* and* S. aurantiacum* were the* Scedosporium* species with a high incidence except for* L. prolificans* [4]. Based on our study, we found that in China the* P. boydii* complex represents the most prevalent species (16/21) followed by* S. apiospermum* (5/21), which is the same as in Northern Spain and France [34, 35]. In the Chinese strains, we could not find* S. aurantiacum*, which had a high incidence in Australia and a relatively low incidence in Europe.

Antifungal susceptibility profiles for clinical breakpoint of* Scedosporium* species are still under study. Of the four antifungal tested in this study, VOR was found to be the most active agent against* Scedosporium* species; POS was the second, and AMB had limited antifungal activity. For ITR, the intraspecific diversity was obvious as the range of MIC from 2 to 32 *μ*g/mL and* P. boydii* had a lower MIC_90_ than that of* P. ellipsoidea* and* S. apiospermum.* This* in vitro* susceptibility data is consistent with previous reports [32, 34, 36–38]. In China, most cases caused by* Scedosporium* were treated with either ITR or VOR. Although* Scedosporium* strains have relatively high MIC values for ITR (GM = 5.25)* in vitro,* studies have verified that they are clinically effective [7].

## 5. Conclusion

The* P. boydii* complex and* S. apiospermum* were the main* Scedosporium* species in clinical samples in China.* Scedosporium* species can be distinguished unambiguously by all the five loci in this study. VOR is the most active agent* in vitro* against the set of* Scedosporium* species in this study, followed by POS and ITR.

## Figures and Tables

**Table 1:** Origin, sequence data, and species identification of studied isolates.

BMU number	Clinical sample	GenBank accession number					Molecular identification
		BT2	CAL	RPB	SOD	ACT	
00488	Unknown	KP981202	KP981130	KP981185	KP981153	KP981107	*P. boydii*
00491^*∗*^	Unknown	KP981214	KP981131	KP981179	KP981154	KP981108	*S. apiospermum*
01112	Unknown	KP981203	KP981132	KP981192	KP981155	KP981109	*P. boydii*
01113	Unknown	KP981204	KP981133	KP981181	KP981156	KP981110	*P. boydii*
01114	Unknown	KP981199	KP981134	KP981193	KP981157	KP981111	*P. boydii*
01115	Unknown	KP981200	KP981135	KP981194	KP981158	KP981112	*P. angusta*
01116	Unknown	KP981201	KP981136	KP981186	KP981159	KP981113	*P. boydii*
01117	Eye ball	KP981205	KP981138	KP981180	KP981160	KP981114	*S. apiospermum*
01118	Unknown	KP981215	KP981137	KP981190	KP981161	KP981115	*P. ellipsoidea*
01272	Sputum	KP981206	KP981139	KP981182	KP981162	KP981116	*P. boydii*
01297^*∗*^	Unknown	KP981221	KP981140	KP981195	KP981163	KP981117	*P. fusoidea*
02948	Nasal sinus	KP981211	KP981141	KP981196	KP981164	KP981118	*P. boydii*
03882	Sputum	KP981207	KP981142	KP981176	KP981165	KP981119	*S. apiospermum*
04111	Joint fluid	KP981219	KP981143	KP981178	KP981166	KP981120	*S. apiospermum*
04729	BALF	KP981216	KP981144	KP981198	KP981167	KP981121	*S. apiospermum*
04730	Nasal sinus	KP981217	KP981145	KP981191	KP981168	KP981122	*P. ellipsoidea*
04772	CSF	KP981220	KP981146	KP981183	KP981169	KP981123	*P. ellipsoidea*
04780	BALF	KP981218	KP981147	KP981187	KP981170	KP981124	*P. ellipsoidea*
05145	Unknown	KP981213	KP981148	KP981189	KP981171	KP981125	*P. ellipsoidea*
07108	Sputum	KP981208	KP981149	KP981188	KP981172	KP981126	*P. ellipsoidea*
07224	Brain	KP981209	KP981150	KP981184	KP981173	KP981127	*P. boydii*
07374	Pus	KP981212	KP981151	KP981197	KP981174	KP981128	*P. boydii*
07462	CSF	KP981210	KP981152	KP981177	KP981175	KP981129	*S. apiospermum*

^**∗**^Strains from Japan.

**Table 2:** * In vitro* susceptibility of 23 *Scedosporium* strains studied in this paper.

Strain species	Strain ID number	MIC (*μ*g/mL)			
		VOR	POS	ITR	AMB
*P. boydii*	BMU 00488	0.25	2	4	4
	BMU 01112	0.25	2	2	32
	BMU 01113	0.25	4	4	32
	BMU 01114	0.5	2	4	8
	BMU 01116	0.5	2	4	4
	BMU 01272	0.5	2	2	8
	BMU 02948	0.5	2	2	4
	BMU 07224	0.25	2	4	16
	BMU 07374	0.25	2	4	4

*P. ellipsoidea*	BMU 01118	1	4	32	4
	BMU 04730	0.5	2	2	4
	BMU 04772	0.5	4	32	32
	BMU 04780	0.5	4	4	16
	BMU 05145	0.5	4	32	8
	BMU 07108	0.25	4	4	8

*P. angusta*	BMU 01115	0.5	2	4	8

*P. fusoidea*	BMU 01297	0.5	2	2	32

*S. apiospermum*	BMU 00491	0.5	4	4	4
	BMU 01117	0.5	4	4	8
	BMU 03882	0.5	2	4	8
	BMU 04111	1	4	32	16
	BMU 04729	0.5	2	2	8
	BMU 07462	1	2	32	8

**Table 3:** The MIC_50_ and MIC_90_ for the four antifungal agents against *P*. *boydii*, *P*. *ellipsoidea*, and *S*. *apiospermum*.

Species (number of isolates)	Drug concentration (*μ*g/mL)							
	VOR		POS		ITR		AMB	
	MIC_50_	MIC_90_	MIC_50_	MIC_90_	MIC_50_	MIC_90_	MIC_50_	MIC_90_
*P*. *boydii *(9)	0.25	0.5	2	4	4	4	8	32
*P*. *ellipsoidea* (6)	0.5	1	4	4	4	32	8	32
*S*. *apiospermum* (6)	0.5	1	2	4	4	32	8	16
